# Systems Approach to Studying Animal Sociality: Individual Position versus Group Organization in Dynamic Social Network Models

**DOI:** 10.1371/journal.pone.0015789

**Published:** 2010-12-23

**Authors:** Karlo Hock, Kah Loon Ng, Nina H. Fefferman

**Affiliations:** 1 Department of Ecology, Evolution and Natural Resources, Rutgers, The State University of New Jersey, New Brunswick, New Jersey, United States of America; 2 Department of Mathematics, National University of Singapore, Singapore, Singapore; 3 Center for Discrete Mathematics and Theoretical Computer Science (DIMACS), Rutgers, The State University of New Jersey, New Brunswick, New Jersey, United States of America; Yale University, United States of America

## Abstract

Social networks can be used to represent group structure as a network of interacting components, and also to quantify both the position of each individual and the global properties of a group. In a series of simulation experiments based on dynamic social networks, we test the prediction that social behaviors that help individuals reach prominence within their social group may conflict with their potential to benefit from their social environment. In addition to cases where individuals were able to benefit from improving both their personal relative importance and group organization, using only simple rules of social affiliation we were able to obtain results in which individuals would face a trade-off between these factors. While selection would favor (or work against) social behaviors that concordantly increase (or decrease, respectively) fitness at both individual and group level, when these factors conflict with each other the eventual selective pressure would depend on the relative returns individuals get from their social environment and their position within it. The presented results highlight the importance of a systems approach to studying animal sociality, in which the effects of social behaviors should be viewed not only through the benefits that those provide to individuals, but also in terms of how they affect broader social environment and how in turn this is reflected back on an individual's fitness.

## Introduction

Fitness of an individual in a social context is affected by its own social behavior and that of its social partners. The interaction patterns produced by such behaviors affect each individual differently, depending on its position within the group structure [1]. However, not all group structures have the same effect on their constituent members. For example, some organization patterns may be better at transmitting information between various group components [2], while others may be better at accumulating and processing it [3]. As it is likely that such differently organized groups will result in the emergence of individuals that will not have identical social roles, either within or across these groups, it follows that fitness of an individual in a social setting will depend not only on its own position within that group's organization, but also on the way the group is organized as a whole.

Within such groups, each individual will also influence the fitness of its partners through its interactions [4]–[6], and the respective interaction pattern of that individual will determine its specific contribution as well as define its personal position in the group. For example, some individuals will have many direct interactions, and will therefore be able to affect more partners more easily [7], [8], while in others having few interactions may in fact be characteristic of dominant individuals [1]. In either case, social behavior of each individual will not only determine its own position within the group, but also contribute to the group structure, and will accordingly affect fitness returns the individual gets from both factors. If the organization of the group as it is defined by the interaction pattern of each individual was not predetermined, such structure can then be seen as the emergent property of the group [9]–[11], arising from individual social behaviors but also providing fitness consequences that cannot be predicted by observing the individual interactions alone.

Social networks have a long and successful history of being used to represent group structure as a network of interacting components [12]–[14]. In biology, those components are individuals, represented as nodes in a network, while their interactions are represented as links, or edges, between the nodes [15]–[18]. The relative importance of each node in a network can then be characterized using various measures of position and connectivity within the network, which are referred to as centrality metrics in network theory [12], [16]. One major advantage of social networks lies in their flexibility: there are many different ways to define centrality, just as there are many ways to define importance in a social group [12]–[14], [16]. Centrality of an individual node in a network depends on its connection pattern, just as, for example, the definition of an alpha individual in a social group depends on its interaction patterns with other group members [19]. Depending on the kind of centrality used, its value can then be related to its immediate neighbors, more distant components of the network, or both. However, in addition to characterizing the relative position of each individual component within the network, social networks can also be used to characterize the overall structure of the group, using group-wide centrality metrics to quantify the level of organization according to some criterion [20], [21]. This ability to combine individual and group properties in analysis makes social networks ideal to study how social behaviors, such as decisions whether and with whom to interact, can lead to distinct selective pressures from both social position of each individual and group organization.

However, testing this paradigm empirically in computational simulation experiments requires several assumptions to link centrality and fitness. First, we will assume that individuals enjoying higher centrality will also be able to benefit more from their social environment. The assumption of advantageous social position could equally well be linked to lower centrality, since we will focus on general trends. In other words, we do not intend to propose a link between high centrality and high fitness in social systems found in animal groups, or indeed that centrality is in any way a determinant rather than a correlate of some fitness benefits immanent in social position [1]–[3]. Rather, we will simply use high centrality as a way to contrast different scenarios used in our simulations. Second, we will assume that individuals will benefit from having important partners, and will thus prefer those over less central ones. Again, even though there are striking examples in animal social systems, such as well-connected elephant matriarchs leading the herd to resources [3] or beta male white-tailed manikins benefiting from connectedness of their respective alphas to females [1], we do not claim that high centrality individuals will be, or should be perceived as, a defining characteristic of desirable social partners in all, or even in the majority of, animal social systems. Finally, we will assume that greater levels of group organization will impart greater benefits to individuals in a group. While an increasing body of evidence suggests that various aspects of colony organization in social insects contributes to the overall colony success [22], [23], distributive decision-making processes could be equally adaptive in other groups [24], [25]. However, for the purpose of our models, social behaviors that lead to greater level of group-wide organization will be treated as if they also improve the fitness of individuals. Thus, the behaviors that produce them will be treated as if being under positive selection in our models, just as would those that improve its personal centrality. In sum, the motivation behind each of the three assumptions is that individuals should behave selfishly and therefore exhibit social behaviors that benefit them regardless of how such benefit is obtained.

Combining these three assumptions outlines a social environment in which it is on one hand beneficial to be central, as this will benefit the individual both directly and by making it a more desirable partner, and on the other it is also beneficial to live in a highly organized, efficient group. To simulate such a social system, we used a dynamic network environment in which we were able to model not only how individual affiliation choices affected the fate of individuals that exhibited them, but also the emergence of group organization from individual behaviors. Thus, in these models the group structure was neither static nor predetermined, and the individuals were freely associating with their partners. Moreover, the only mechanism that influenced their social choice on whether to remain connected to particular partners was based on the perceived importance of those partners. The social dynamics in these systems was therefore based on dropping connections to some partners, those perceived as less desirable social partners, and randomly adding new partners of unknown desirability.

To explore a potential diversity of emergent group structures that arose from this simple drop/add process, we used three different centrality criteria to guide affiliations of individuals in a network based on directed connections: Popularity (P; also referred to as in-degree), Closeness (C), and Betweenness (B) (*sensu* Freeman [12]). These three measures of relative importance were then used to evaluate their social partners and guide their social choices, yielding P-networks, C-networks, and B-networks, respectively. The results from each of those were then compared to those obtained from random affiliation networks, or R-networks, in which individuals were unable to assess the centrality of their existing affiliations, and therefore dropped connections at random rather than dropping those perceived to be the least desirable partners. While the mathematical details of these centrality measures, as well as the exact rules of how dynamic directed networks were constructed, are given in the Methods section, it is important to note that in our simulations we do not assume that any of these metrics is a direct analog of biological fitness in all, or indeed in the majority of, social contexts. Nevertheless, depending on the social context in which it is observed, i.e. what interactions in a network represent, these metrics can, and have been, used to identify individuals that would appear to benefit more or less from the social environment [1], [2], [16]–[18]. Therefore, these measures can be used as correlates of social components of fitness under certain conditions, and their use to describe biological systems is not unprecedented. For example, high Popularity could characterize individuals that receive many behaviors directed at them, such as grooming [26]; high Closeness can define individuals that are able to easily and rapidly reach others in a group, but also rapidly transmit infectious agents [7], [8]; and high Betweenness can be viewed as the network position of an individual that characterizes it as a necessary intermediary or ‘information bottleneck’ in a group or population [2]. Moreover, these metrics differ conceptually in the way in which individuals can become more desirable partners when connecting to others in networks where connections are directed, i.e. where both initiator and target of each social connection are known: Popularity enhances the desirability of partners while doing nothing for the actor; Closeness has no effect on either actor or recipient; and Betweenness generally enhances the desirability of both actor and recipient of the connection. While there are many other metrics that can also be used in such studies [12]–[18], for this initial study these metrics provide sufficiently diverse examples of how importance can be defined in a group.

**Figure 1:**
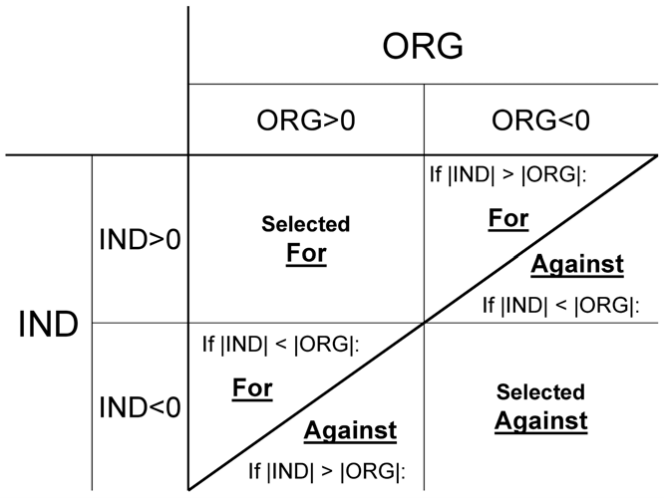
Conceptual representation of selective pressure outcomes for social behaviors based on their potential fitness consequences. Fitness an individual gets from its social environment will depend not only on its own individual position within the group (IND), but also on the way the group is organized as a whole (ORG). When the effects of both factors increase the fitness of an individual, the driving social behavior is under positive selective pressure. When both factors decrease fitness, the behavior is counter-selected. When these factors are in conflict, their relative magnitude will determine the net selective pressure, and the factor with a greater relative magnitude will determine the direction of selection for such behavior. When both factors have the same magnitude but are in conflict with each other, their effects will be at equilibrium and the behavior will be under stabilizing selection.

In very basic terms, as social behaviors change the interaction pattern of an individual in a group, they will have either positive or negative consequences for its personal social position in a social structure of that group. Similarly, they will also positively or negatively affect the group organization. When these effects are then combined, we can distinguish four different outcomes in which these factors can be concordant with or conflict with each other (Figure 1). While selective pressures will be synergistic when social behaviors concordantly increase (or decrease) fitness by improving (or compromising) both individual position and group organization, there could also be cases when the directions of these selective pressures will be in conflict. Certain behaviors may increase the fitness of an individual by making its social position more advantageous while at the same time decreasing the viability of, and the benefits this individual receives from, its social environment. Alternatively, some behaviors could increase the efficiency of group organization while preventing individuals from maximizing their potential for advantageous personal position in a group. In the latter case, the individuals may then profit indirectly from having particular partners in a group, such as partners that happen to be very central in some way, and without necessarily becoming central themselves (e.g. a member of a herd led by a successful matriarch, as in McComb et al. [3]). The resulting overall direction of selective pressure would therefore depend on the relative magnitude of these effects (cf. Figure 1). Demonstrating the possibility of obtaining such outcomes in a simulated environment, with only very simple affiliation rules directing social choice, will help raise awareness about and inspire studies to examine indirect effects of sociality as they relate to the properties of a system as a whole.

## Results

Before being able to determine whether social behavior can lead to concordant or conflicting tendencies between improving personal social position and group organization, we define the basic criteria of success in our simulations. As high centrality is used in our simulations to be analogous to high utility of sociality, we used both an individual's ability to attain high centrality and maintain it afterwards as a criterion for personal success. Group success was correspondingly defined as a high level of group-wide centrality in the simulation run. As the results of group organization were identical to those of Fefferman & Ng [21], they are not explicitly reported here but rather used for comparison purposes. For methodological details on how to define and determine group-wide centrality, readers are thus referred to their exhaustive descriptions in Fefferman and Ng [21].

**Figure 2:**
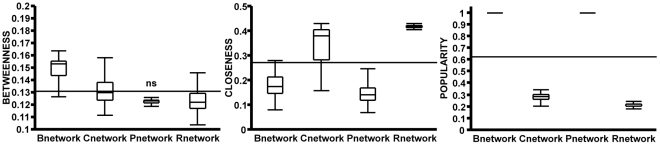
Comparing maximum attainable centrality across networks. With the exception of B-centrality in P-networks (labeled as *ns*), all affiliation preferences raised values of P- and B-centrality metric above those recorded in R-networks. In contrast, none of the affiliation-driven networks were able to raise the values of C-centrality above those recorded in R-network. The box plots indicate descriptive statistics: median, quartiles, maxima and minima; horizontal line in each graph indicates the grand mean for the respective centrality metric.

When the ability of an individual to attain high centrality in a network was used as a criterion for its success in a group, individuals in affiliation-driven networks generally fared better than in R-networks (Figure 2). With the exception of P-networks not raising the B-centrality value above those obtained from R-networks, affiliation-driven networks were better at improving both P-centrality and B-centrality (B-centrality χ^2^(3,N = 1200) = 513.11, p<0.0001; P-centrality χ^2^(3,N = 1200) = 1137.7, p<0.0001; p<0.05 for all multiple comparisons with R-network values except B-centrality in P-networks). Nevertheless, highest values of C-centrality were recorded in R-networks (C-centrality χ^2^(3,N = 1200) = 943.54, p<0.0001; p<0.05 for all multiple comparisons with R-network values). Affiliation preferences therefore increased the maximum attainable personal P- and B-centrality in networks. However, this was not a specific phenomenon, as all affiliations, and not just those using P- and B-centrality to guide social choice, elevated these measures. In contrast, maximization of attainable C-centrality occurred when no affiliation preference was used to guide social choice.

Networks significantly differed in the ability of individuals to keep on increasing their centrality rank (B-centrality F_3, 1196_  = 13.13, p<0.0001; C-centrality F_3, 1196_  = 3.75, p = 0.01; P-centrality F_3, 1196_  = 30.27, p<0.0001) (Table 1). While individuals in P-networks experienced significant upward trends for all centrality metrics with respect to the R-networks, those in the B-networks were able to do so only for B- and P-centrality ranks. In contrast, individuals in C-networks did not exhibit any such tendencies. This suggests that individuals were not only able to attain higher success by using B- and P-centrality to guide social choice, but also that significantly more of them would experience increasing trends to improve their relative importance than in R-networks. While this also suggests that some individuals were dropping in rank, the incentive to use affiliation rules was still higher for the listed networks than if no affiliation preferences were used.

**Table 1:** The number of individuals per network type that satisfied the criterion of substantial upward trend in centrality.

	Network Type			
Centrality Metric	B	C	P	R
**B**	**344**/222*	**207**/152	**372**/219*	**208**/154
**C**	**221**/153	**178**/141	**238**/169*	**188**/140
**P**	**300**/190*	**217**/156	**329**/200*	**209**/160

To satisfy the criterion of substantial upward trend in centrality, individuals had to increase their relative centrality rank 7 or more (out of 9) times between successive time intervals for a given centrality metric. The results indicate the total number of individuals (in bold print) that satisfied the criterion/number of networks in which at least one individual that satisfied this criterion was recorded. Non-random networks in which the average number of individuals per network was significantly different from the respective R-networks in multiple comparisons are denoted with

*.

**Figure 3:**
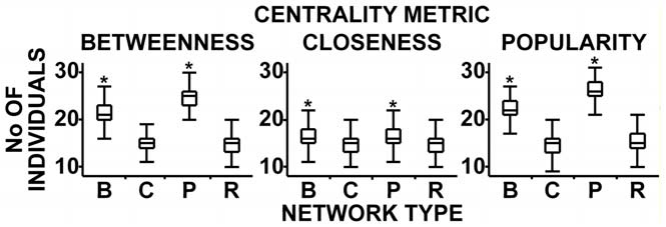
Comparing individuals that successfully maintained increased centrality rank for 7 or more time intervals. B- and P-networks had significantly more such individuals than R-networks for all metrics. C-networks were indistinguishable from the R-networks. Metrics that were significantly different from those in R-networks in multiple comparisons are denoted with *. The box plots indicate descriptive statistics: median, quartiles, maxima and minima.

To determine whether individuals could expect to maintain increased personal centrality while social dynamics processes continue, we compared the numbers of individuals consistently maintaining higher than initial rank in affiliation-driven networks to those observed in the R-networks (Figure 3). Significant differences between networks were recorded for all centrality metrics (B-centrality F_3, 659.91_  = 1480.32, p<0.0001; C-centrality F_3, 1196_  = 32.19, p<0.0001; P-centrality F_3, 657.05_  = 1980.75, p<0.0001). In both B- and P-networks, significantly more individuals per network consistently maintained higher than initial ranks than were observed for R-networks. The expectations in C-networks were comparable to R-networks. As, individuals in B- or P-networks were able to maintain B- and P-centrality ranks that were higher than their initial ranks, they were able to remain more central for extended periods of time (while no such trends were detected for C-networks). The affiliation rules thus allowed them to keep their elevated importance in a group once it was achieved, and enjoy any potential benefits that could come with it. As in B- and P-networks both B- and P-centrality were consistently increased, the correlation between the two metrics would suggest that cues for one metric could be used as a reliable proxy of the other [21]. When these results on individual centrality are taken together, we conclude that individuals were able to successfully raise their P-centrality in either B- or P-networks, that they would do best to raise their B-centrality in a B-network (or perhaps P-network if stability of elevated social position was more important than attainable maximum), and that none of the affiliation-driven networks were useful for raising C-centrality, the values for which were the highest in R-networks.

Most interestingly, whether the relative centrality results of individuals in modeled networks were in concordance with or in contrast to the increasing trends of group-wide centrality measures (as reported in Fefferman and Ng [21]) was dependent on the network type (Table 2). In P-networks, the expected increase of personal P-centrality of individuals was concordant with higher group-wide P-centrality. This suggests that social behavior based on P-centrality would experience positive selective pressure from both factors, as follows from assumptions of advantageous high centrality we used in our models. Conversely, no network was more successful at increasing C-centrality either at individual or group-wide level than the R-network (with any positive trends in P-networks being potentially offset by the negative expectation to maintain increased rank). This suggests that attempts to maximize C-centrality would not benefit from using C-centrality of potential partners as an affiliation cue, and that such behaviors would be selected against. Unlike the previous two centrality metrics, groups attempting to maximize B-centrality would face conflicting influences at the level of individual and group-wide B-centralities. Personal B-centrality will be increased in B- (and potentially P-) networks, but the highest levels of group-wide B-centrality will be achieved in C- and R-networks. This would therefore result in conflicting pressures on individuals in these networks, and the selective pressure on such behaviors would therefore result in a trade-off between individual and group-wide returns, ultimately depending on the relative magnitude of the returns from each effect. Thus, using just simple affiliation behaviors in a network of socially defined, but otherwise identical, individuals, we were able to replicate all hypothesized outcomes from Figure 1.

**Table 2:** Comparison of individual and group-wide results.

	Network of Choice			
Metric to Increase	Group-wide	Individual	Concordant?	Pressure to organize
B	C, R	B (or P)	NO	?
C	R	R	YES	NEGATIVE
P	B, P	B, P	YES	POSITIVE

Maximization of B-centrality was ambiguous, with conflicting pressures. C-centrality maximization was under uniform pressure that did not favor organization. P-centrality maximization was under uniform pressure to organize using either B- or P-networks.

## Discussion

Fitness of an individual is inextricably linked with the characteristics of its social environment. However, not all individuals in an interacting group of individuals will have the same role or relative social position [27]. The relative importance and social position of each individual within its group will in turn depend on the attributes of its partners and the pattern of its interactions [1]–[3]. The contributions of sociality to fitness, i.e. the extent to which interactions with other individuals affect the fitness of a focal individual, will therefore depend both on interaction partners that comprise individual's social environment and its relative position within that environment. The associated pressures should then determine whether behaviors that lead to beneficial social organization will be favored by selection. The results of our simulation experiments demonstrate that it is possible to get either concordant or conflicting pressures between group organization and individual profiling, even when using simple affiliation rules to guide social choice.

In animal populations, highly complex social organization can result from a uniform set of very simple operational rules [9], and such algorithms have been applied to diverse questions like the nest-site selective algorithms of ants [28], population persistence of mammals [3], [29], [30], long-term reproductive success in birds [1], and self-organization of primate groups [11], [31]. However, the effect that group organization, as an emergent property, has on individuals received surprisingly little attention to date. Evolutionary theory predicts that behaviors that increase fitness by any mechanism should be favored by selection [32]. With regard to fitness consequences that stem from sociality, selective pressures may act to increase the direct gains an individual expects to attain in a group in relation to its peers, and/or to increase indirect, distributive benefits conferred to it by the successful organization of its social environment. If indirect benefits to all individuals in a group arise from the organization of a group where social connections become concentrated in a few individuals, as is observed e.g. in social networks of African elephants [3], the selective pressure would favor the emergence of a group-wide centrality built around these key individuals. Conversely, if benefits stem from a distributive decision-making system, as is the case in many social groups without a prominent central individual [24], [25], selection will favor low centrality at the group level, even though it might be beneficial for individuals to be personally central. As long as indirect benefits from such arrangements outweigh any costs to individuals, such as loss of personal centrality in either centralized or decentralized groups, the mechanisms that favor such arrangement will be under positive selective pressure.

Social behaviors that benefit individuals by concordantly increasing the likelihood that they will be favorably positioned in their group and the benefits they get from membership in their respective group will clearly be favored by selection. These conditions are well illustrated by the results of P-networks, where, in their attempts to achieve high P-centrality in a simulation, individuals were able to both increase relative personal P-centrality and also use the same set of rules as the best option to maximize group-wide P-centrality. Thus, if increased P-centrality was associated with fitness benefits from sociality, as was assumed in our models and as it could also be the case in some biological networks (such as those based on allogrooming [26]), groups organized as P-networks would be successful at increasing individuals' fitness both directly and indirectly.

In contrast to such synergistic positive effect, social affiliation behaviors could also result in conditions where returns from both personal position and group-wide organization are concordantly negative. In such cases, behaviors that lead to this kind of result will be under a negative selective pressure. Networks based on C-centrality affiliation preferences are an example of just such conditions. Not only were the individuals unable to experience levels of relative personal centrality beyond those in randomly organized networks, but also the group-wide C-centrality was at its greatest under the same randomized circumstances. Assuming that C-centrality was associated with high fitness, we expect that individuals would be under negative selective pressure to use C-centrality as a cue for social affiliations and group organization. Thus, and somewhat counterintuitively, individuals should be either oblivious to the cues that signal high social fitness when choosing partners, or even be primed to actively avoid these cues. Conversely, if it was in fact low C-centrality that was analogous to increased fitness, individuals could use organizational rules that would hamper the emergence of high C-centrality individuals in a group (perhaps also ensuring that they themselves would not emerge as one), as was observed in our affiliation-driven networks. Given that C-centrality involves individuals aiming to be connected to other individuals in the shortest number of steps, such mechanisms could potentially be interpreted as interacting groups organized in a way that slows the spread of disease and eliminates the emergence of ‘superspreaders’ in a group [7], [8], [33].

Selecting to affiliate with partners of particular quality could also increase the benefits that individuals get from their social environment. Using a hypothetical example, if individuals benefit by preferring to affiliate with partners that accumulate information through their numerous social connections, they would likely also benefit from group organization that favors greater overall accumulation of information in at least some individuals. However, it is intriguing that there could be behaviors which would help individuals accumulate more information at the expense of group-wide information accumulation. Alternatively, this could also mean that the indirect benefits from group organization and social partners could compensate for seemingly suboptimal position of an individual in a group. Maximization of B-centrality provides an example of such conflicting influences at the individual- and group-level. Being connected to the high quality intermediaries increases the personal quality of the individual as an intermediary [34]. Accordingly, personal B-centrality was increased in B-networks. However, the group-wide increase of B-centrality was the greatest in C- and R-networks. If high B-centrality is assumed to be analogous to increased fitness, the network of choice would therefore be unclear. This example could reflect situations in which the benefits of having only a few individuals that act as ‘brokers’ within or between groups [2] is contrasted with distributive information processing through consensus decisions [24], [25]. In such cases, social behaviors result in a trade-off between direct personal benefits and advantageous social organization. Therefore, the eventual selective pressure on exhibited social behaviors will depend on the relative magnitude of the returns the individuals get from these two factors.

The biological examples supporting these tradeoffs abound, both in the terms of experimentally determined parameters and theoretical considerations. Groups of social animals may be organized so as to maximize the centralized [35] or distributive, consensual decision-making processes [25]. Concentrating benefits that can be accrued through social interactions in particular members of a group may therefore lead to an increased fitness for other participants [2], [3], [36], and as a result their emergence may be favored by selection and indirectly supported by behaviors of all group members. Conversely, these benefits may be offset by the increased risks for such individuals, for example from metabolic costs of dominance [37], [38], or risks they pose to others, such as their increased propensity to transmit infectious agents [7], [8], [33] or disrupt reproduction through hyper-aggressiveness [39]. Furthermore, the interaction patterns in some cases may be used by animals to minimize the impacts of an unfavorable social situation, and individuals may benefit from social organization even though they themselves do not occupy the most prominent social roles in their groups. For example, even non-central individuals benefit from group organization when it is based on directed allogrooming behaviors [26], or when it primes the actor for a future, more successful social role [1]. Finally, efficient social organization may affect the direction of selection for the participants. For example, while centralization may be accompanied by increased cognitive capacities that allow for management of this social information [40], increased efficiency of group organization may in fact allow for a reduced sophistication and connectivity of individuals in a group [41].

While both personal social position and social organization of a group benefit individuals embedded in such groups, it is intriguing to interpret these results from the multilevel selection standpoint. If the emergence of efficient group organization is dependent on the behavioral phenotype shared by at least a portion of individuals that constitute a group, as is the case with trait-group concept [42], group organization could be viewed in the context of emergent properties characterizing groups. Theoretical considerations backed by recent empirical evidence would suggest that organization of animal social groups may be key to their success [22], [43]–[45]. However, in contrast to the efforts where fitness is evaluated over generations [46], [47], our results demonstrate how differences in survival can instantly arise from social behaviors, and do so even if all individuals exhibit only a single, simple, and non-plastic phenotype. Moreover, while benefits to the individual are key to evolutionary success, competition to improve personal position within a group may negatively affect others, leading to decreased viability of social environment. The success of individuals embedded in social groups may therefore depend on their personal prominence as well as their effect on other group members, and fitness will be maximized in individuals that are best able to balance these factors. Thus, some social environments may provide individuals with advantages over other, differently organized groups. Again, using the hypothetical information accumulation example, groups that are able to accumulate more information due to their structuring will be able to outcompete other, less efficiently organized groups. More detailed studies on particular mechanisms of how this could occur are currently ongoing (Hock and Fefferman, in preparation).

In our models, however, group-wide benefits are not simply equivalent to average group benefits. Group organization in our simulations was treated as an emergent property that stems from individual social behaviors. Thus, it is not a simple average of centrality of individuals, but rather a derived characteristic of a group. As such, it is possible that individuals will suffer negative selective pressure from inefficient group organization, and thus decrease net fitness of individuals not just in comparison with other groups, but also without any intergroup competition. In other words, certain actions that are advantageous to an actor may also diminish the fitness of everyone else in the group, ultimately decreasing the fitness of everyone involved: including themselves. Hyper-aggressiveness may be such example [39], in which individuals exhibit behaviors that benefit them under some circumstances, e.g. increase their own mating opportunities, but are ultimately detrimental, decreasing the number of offspring an individual would be able to produce if no group member behaved in this way. Thus, while some behaviors may be individually adaptive, they could be disruptive in a broader social context. Competition among colonies could be another example where such considerations could be important, for example in social insects where the importance of colony structure could determine success in competition among colonies [22].

In sum, our results highlight the importance of a systems approach to studying animal sociality. When considering fitness of individuals in a social context, the focus has traditionally been on studying how successful each individual is within its respective interaction group. We propose that equal attention should be devoted to also consider how an individual's actions affect its social environment and how this in turn will be reflected back on its personal fitness. The ability of social networks to quantify both individual position and group organization promises new and exciting ways how these powerful tools could be used to study animal sociality. By looking at both direct and indirect benefits individuals receive from the social context within which they are embedded, and by taking into account personal position as well as emergent properties of a group, the current study provides a quantitative framework for future studies of social organization of animal groups.

## Methods

### Overview

Few analytical tools provide such a breadth of opportunities to study the structure of social systems as social network analysis. Social systems are often complex, and the overall social environment is a product of many individual decisions and behaviors exhibited by its constituent members. To understand the fundamental principles of how social structure emerges from individual actions, we need methods to quantify the outcome both for each member of a social system and the functioning of a system as a whole. Social networks offer just such an analytical framework by quantitatively characterizing both the positions of individual entities and the overall structure of the network.

Using dynamic network models in a simulated social group, it is therefore possible to demonstrate that the social behavior of individuals may either aid or disrupt organization of the group. Just as an individual's decisions impact its direct social partners and its social environment in general, its behavior will affect its own position in its local social group, as well as its position in the overall social structure. We will use a simple example to illustrate various outcomes of individual behavioral decisions on both the individual and the wider social environment. Under the assumption that such advantages can be described using network metrics, we designed a simulation study to test whether behavioral decisions aimed at improving some measure analogous to a certain social position also necessarily positively affect the overall organization of a social group, or whether some behavioral strategies may in fact lead to conflicting outcomes between these effects. While we in no way mean to suggest that biological fitness is directly analogous to network centrality, we hope that demonstrating the generality of such paradigm even in such simple context will stimulate future research in this area, especially the often overlooked aspect of looking at a social group as a system with emergent properties that cannot be predicted from individual components alone.

### Social networks and centrality

To examine the impact of individual choice in dynamic social affiliation on the social success of the participating individuals, we built upon the series of models presented in Fefferman and Ng [21] in which individuals constantly re-evaluated the relative ‘quality’ of their affiliates and maintained or discontinued these affiliations accordingly in social groups resembling small world networks [48]. Though many different centrality metrics exist in social network analysis (each capturing different aspects of social position or organization within a connected social group [13], [14], [16]), we chose three basic, traditional centrality measures broadly used in the field of social network theory. The social quality of an individual in the presented models was thus contingent on one of the three different centrality measures [12]: (1) Popularity (P; also called in-degree), (2) Closeness (C), and (3) Betweenness (B). These particular metrics were chosen without intention to imply any potential importance in characterization of animal groups: indeed, a wide variety of social network metrics exists and the list of useful parameters continues to grow. Rather, their simplicity and historical importance in social network theory made them intuitive candidates to demonstrate the potential range of relative outcomes that are possible in a social system. More specifically, rather than focusing on what these metrics may or may not characterize in any given social system or animal group, we used them to investigate general trends and directionality of such trends that socially embedded individuals may encounter. Though for modeling purposes we assumed that high centrality at both individual and group level was advantageous in terms of payoffs individuals would get from sociality, the situation may well be completely opposite in some animal social systems. The general nature of our models does not preclude such contrasting conclusions: it simply highlights the possibility of observing different combinations of individual and group-wide trends, as well as the potential importance of, often overlooked, benefits individuals could get from their wider social environment.

In the modeled interacting groups, individuals were represented in a digraph environment, where connections between individuals specified not only the identity of the connected individuals, but also the directionality of interactions, identifying which individuals were the initiators of the recorded social affiliations. Individuals dynamically evaluated the relative levels of the three centrality measures that characterized them as more or less attractive social partners, and used these measures to guide their decisions in social affiliation. While individuals preferred to maintain affiliation with individuals exhibiting comparatively high values of a particular centrality measure, we in no way mean to suggest that these three measures (from among many defined already by social network theorists to examine different facets of organizational success) are by themselves advantageous for fitness, or of any particular importance in choosing social partners in any particular animal social system. They are, however, easily distinguishable and sufficiently diverse so as to demonstrate a variety of ways in which individual actions may or may not result in concordant individual- and group-level outcomes.

### Defining network environment and centrality metrics

To explore the distribution of centrality values in an interacting group under each of the dynamic affiliation strategies, we designed a directed graph (or digraph) environment. Each node in the graph represents a particular individual in the network and a directed edge from one vertex to another represents a directed connection (or affiliation) between the pair of individuals. More precisely, if *G* is the digraph, we let the set *V*(*G*)  =  {*v*_1_, *v*_2_,…, *v_n_*} to be the set of nodes and *E*(*G*) to be the set of edges in *G*. We say that *v_i_* is adjacent to *v_j_* (or *v_j_* is adjacent from *v_i_*) if there is an edge in *G* from *v_i_* to *v_j_*. In this case, *v_j_* is said to be an out-neighbor of *v_i_*. The in-degree of a node *v_i_*, denoted by *d*_in_(*v_i_*), is the number of nodes in *G* that has *v_i_* as an out-neighbor. The distance between nodes *v_i_* and *v_j_*, denoted by *d*(*v_i_*,*v_j_*), is the length of the shortest path from *v_i_* to *v_j_* in *G*. If there is no path between *v_i_* and *v_j_* in *G*, we set *d*(*v_i_*,*v_j_*) to be equal to *n* defined as the number of nodes in *G*.

The three widely used measures of centrality from social network studies are defined as follows:

- The Popularity centrality measure of a node *v_i_*, denoted by *P*(*v_i_*) is defined to be 
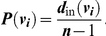

  Note that the maximum value of *P*(*v_i_*) is 1 when every other node of *G* has *v_i_* as an out-neighbor. As such, Popularity (that is, in-degree) measures the number of social connections that connect to the focal individual in a group.
- The Closeness centrality measure of a node *v_i_*, denoted by *C*(*v_i_*) is defined to be 
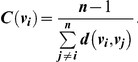

  Note that the maximum value of *C*(*v_i_*) is 1 when every other node of *G* is an out-neighbor of *v_i_*. As such, Closeness measures the number of steps needed for the information originating from the focal individual to reach every other individual in a group.
- Let *S* be the set of all shortest paths between all pairs of nodes in *G* and *count*(*v_i_*) be the number of shortest paths in *S* that contains node *v_i_* as an intermediate node. The Betweenness centrality measure of a node *v_i_*, denoted by *B*(*v_i_*) is defined to be
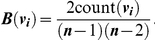

  Note that the maximum value of *B*(*v_i_*) is attained when *v_i_* is a necessary intermediary that connects all pairs of two other nodes in a network via the shortest available route. As such, Betweenness measures the number of shortest paths between all pairs of other individuals that include the focal individual.

From the aspect of biological analogues, Popularity can therefore be viewed as the self-reinforcing ability to attract social connections, Closeness as the ability to influence other individuals in a group and the ease of doing so, while Betweenness describes how well an individual is positioned to act as a necessary intermediary in a group or population. While biological analogues of these centrality measures can certainly be envisioned, the association between social importance and particular centrality metric will likely be specific for the social system in question. In other words, high Popularity will mean advantageous social position in one social system or species, and characterize something completely different, or indeed opposite, in another. It is therefore not our intention here to equate the chosen centrality metric with a particular context or a particular taxon. Rather, we use these metrics to demonstrate the as-of-yet untapped potential to use network theory to study emerging social complexity in animal groups. With successful illustration of these principles, it is our hope that their generality will stimulate future research in specific systems which will then utilize metrics that characterize socially (dis)advantageous positions specific relevant to the biological example in question.

### Simulations

The simulations of the models were developed using Java programming language (Java 1.6.0 API). The preference of each individual *v_i_* for a certain measure of partner centrality was determined at the outset of computation and all individuals in a given network had the same preference (i.e. only a single phenotype) which remained unchanged throughout the simulation (i.e. no phenotypic plasticity). An affiliation network was therefore called a B- (resp. C- and P-) network if it was comprised of B- (resp. C- and P-) individuals that all attempted to maintain connections with partners with the highest B- (resp. C- and P-) centrality. Each simulation started at iteration *t* = 1 when a network (B-, C-, or P-) was initialized. Initially, each individual *v_i_* was assigned five out-neighbors at random, and in each subsequent iteration *v_i_* changed its set of out-neighbors by dropping its connections to two of the five existing out-neighbors and replacing them with two others. Which two connections were dropped by *v_i_* depended on *v_i_*'s affiliation preference, i.e. its network type. For example, if *v_i_* was a B-individual, then the B-centrality of *v_i_*'s five current out-neighbors were ranked and *v_i_* removed its affiliation to the two current out-neighbors with the lowest values of B-centrality. The two replacement out-neighbors were then chosen at random from all other individuals in the network, with the restriction that the two individuals just dropped could not have been re-chosen as replacements during the same iteration. In addition to the B-, C- and P-networks, where individuals changed their out-neighbors according to their prescribed preferences, we also defined an R-network (comprised of R-individuals) to be one where each individual both dropped and replaced two out-neighbors during each iteration completely at random. Such R-networks served as a null-model and provided the means to compare the relative success, and consistency of success, for individuals in the affiliation-driven B-, C- and P-networks.

As well as the models themselves, the simulation parameters were designed so as to be comparable to those from Fefferman and Ng [21]. Each simulation ran for *t* = 200 iterations, and a total of 300 simulations were performed for each network type to ensure the size of the sample was adequate given the stochastic nature of the simulations. For each individual the values of B-, C-, and P-centrality were computed at every iteration *t*. Though each measure of centrality provided a numerical value for each individual's status at each iteration, the numerical values of centrality metrics are highly contingent on properties specific to the actual setup, such as group size [13], [14]. As the primary focus of this demonstration was to compare relative success of individuals in a generalized network and not to test a particular set of conditions, measures associated with each individual were ranked for each network and at every iteration *t* (from the largest to the smallest, allowing for ties if two or more individuals had the same centrality values) with respect to the centrality of all other individuals in a network. This allowed us to determine the relative value of each individual's centrality at every iteration. We also assumed that immediate short-term fluctuations in rank position should be less important than longer-term averages in relative status when stability is concerned. As a result, we averaged the ranks of individuals over intervals of 20 iterations, yielding *T* = 10 time intervals from each experiment over which we calculated rank-average values. This resulted in a composite measure describing the centrality position an individual held in a group for a given period of time and with it also its success in obtaining the expected returns from continued membership in a stable, organized social group.

### Determining personal centrality maximization success

Assuming that high centrality corresponds to high fitness, we set out to determine whether individuals would experience increased expectation to gain from sociality by using social organization rules based on free association. We therefore evaluated whether or not each organizational rule resulted an increase in relative standing in the group.

We first evaluated the maximum attainable centrality for each chosen metric and within each network type. Individuals were ranked according to their absolute centrality, and the centralities of the highest ranking individuals in each group were then compared across networks. For each centrality metric comparison, we used the highest ranking individual for that particular metric, e.g. to compare which rules are the best for generating high P-centrality, we used the highest ranking P-centrality individual from each network. The results were then analyzed using Kruskal-Wallis test, and multiple comparisons with R-network values were performed according to Siegel and Castellan [49].

We then evaluated how many individuals were able to enjoy elevated social position. Success of an individual in an interacting group was linked to its ability to attain a lasting increase in relative centrality rank, and to continuously increase its centrality rank relative to other individuals in a group. To determine whether individuals could expect to experience increased rank in the course of the social organization, we recorded whether an individual was able to consistently maintain a relative rank that was higher than the one it held in the initial time interval of a simulation (for >7 out of 9 time intervals). The average number of individuals in non-random networks that were able to maintain higher than initial rank under the same criteria was then compared with the numbers obtained from R-networks. We recorded these values independently over time intervals, i.e. the sequence of intervals during which an individual held an elevated rank did not matter. This enabled us to assess the consistency in maintaining increased rank regardless of the number of separate occasions during which individuals were able to do so. The obtained values were transformed using square root transformation [50], and then analyzed using ANOVA followed by Tukey's HSD test for comparisons of C-centrality, and Welch ANOVA followed by Games-Howell test for comparisons of B- and P-centrality due to unequal variances between groups.

To examine whether individuals in a given network could expect to experience substantial upward trends in centrality, we recorded the number of times an individual increased its relative centrality rank between successive time intervals. We further recorded the average number of individuals per network that experienced rank increase between successive time intervals 7 or more (out of maximum of 9) times, and compared the results from non-random networks with those obtained from the R-networks to evaluate the effects of affiliation strategies on increased expectation of rank improvement. The obtained values were again transformed using square root transformation, and then analyzed using ANOVA (variances among groups were homoscedastic) followed by two-tailed Dunnett's *q* tests for comparisons against the R-network results.

To determine whether individual- and group-level outcomes were acting on the fitness of individuals in either conflict or concordance, the expected personal centrality success of individuals was then compared to group-wide centrality measures for the same scenarios previously published and described by Fefferman and Ng [21].
